# An Enhanced Privacy-Preserving Authentication Scheme for Vehicle Sensor Networks

**DOI:** 10.3390/s17122854

**Published:** 2017-12-08

**Authors:** Yousheng Zhou, Xiaofeng Zhao, Yi Jiang, Fengjun Shang, Shaojiang Deng, Xiaojun Wang

**Affiliations:** 1 College of Computer Science and Technology, Chongqing University of Posts and Telecommunications, Chongqing 400065, China; zhouys@cqupt.edu.cn (Y.Z.); jiangyi@cqupt.edu.cn (Y.J.); shangfj@cqupt.edu.cn (F.S.); 2 College of Computer Science, Chongqing University, Chongqing 400044, China; 3 School of Cyber Security and Information Law, Chongqing University of Posts and Telecommunications, Chongqing 400065, China; 4 School of Electronic Engineering, Dublin City University, Dublin, Ireland; xiaojun.wang@dcu.ie

**Keywords:** vehicle sensor network, authentication, V2V, provable security

## Abstract

Vehicle sensor networks (VSNs) are ushering in a promising future by enabling more intelligent transportation systems and providing a more efficient driving experience. However, because of their inherent openness, VSNs are subject to a large number of potential security threats. Although various authentication schemes have been proposed for addressing security problems, they are not suitable for VSN applications because of their high computation and communication costs. Chuang and Lee have developed a trust-extended authentication mechanism (TEAM) for vehicle-to-vehicle communication using a transitive trust relationship, which they claim can resist various attacks. However, it fails to counter internal attacks because of the utilization of a shared secret key. In this paper, to eliminate the vulnerability of TEAM, an enhanced privacy-preserving authentication scheme for VSNs is constructed. The security of our proposed scheme is proven under the random oracle model based on the assumption of the computational Diffie–Hellman problem.

## 1. Introduction

With the rapid development of the intelligent transportation systems (ITSs) [1], vehicular ad hoc networks (VANETs) have become increasingly popular. The vehicles in VANETs can communicate with each other via wireless communication [2]. If vehicles can interact with other vehicles or the roadside infrastructure to exchange collected data for decision-making and safer driving, traffic jams can be avoided and the safety of drivers can be guaranteed to the utmost extent; consequently, VANETs are a promising means of improving traffic safety and management. At present, vehicles are equipped with various sensors that can provide valuable data. Further equipping vehicles with onboard sensing devices can turn VANETs into vehicle sensor networks (VSNs) [3]. Therefore, the authentication protocols used in VANETs can also be used in VSNs. Moreover, dynamic traffic information and many types of physical data associated with traffic distributions can be sensed and collected by such vehicular communication networks. Therefore, VSNs are expected to significantly facilitate future wireless communication.

**Figure 1:**
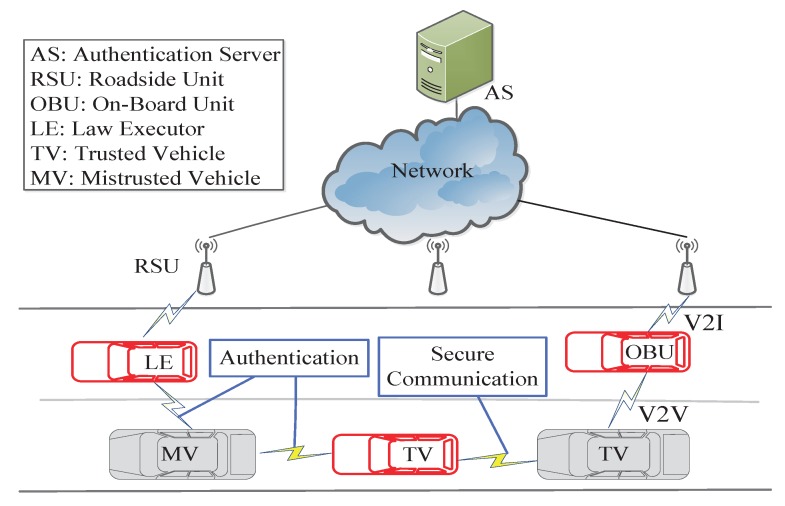
Structure of a vehicular ad-hoc network.

Two types of communication exist in VANETs, namely vehicle-to-vehicle (V2V) communication and vehicle-to-infrastructure (V2I) communication, which depend on two essential kinds of components: onboard units (OBUs) and roadside units (RSUs). As shown in Figure 1, OBUs are the wireless communication units equipped on vehicles, whereas RSUs are wireless access units located at significant places on the road. Generally, to assist vehicles and RSUs in performing certain tasks, such as authentication, a backend server should be deployed remotely. The characteristics of VANETs include self-organization, channel-opening behavior, and rapidly changing and multiple-hop topologies. Due to these characteristics, VANETs are more susceptible to malicious attacks. Since safety and privacy are a concern in many applications in VANETs [4,5], communication security issues are worthy of attention. Among the various security mechanisms used in VANETs, authentication is one basic component that is critical for ensuring security. However, a desirable authentication scheme must be efficient and practical for use in fast-moving scenarios, which means that the computation cost for authentication should be as low as possible to enable real-time response. In addition, privacy preservation should be considered, including the identity privacy, location privacy, and interest privacy. Moreover, the location of a vehicle is closely related to who is driving it. When a vehicle communicates with others in a wireless network, it will not be acceptable to the public if the vehicle’s identity and location are disclosed. Thus, privacy preservation must be achieved in the authentication procedure. In addition, it should be possible for the real identities of the malicious vehicles to be revealed by the authorities when necessary [5]. These requirements pose a considerable challenge for the development of an ideal authentication scheme.

The contributions of this paper are as follows: (1) an enhanced privacy-preserving authentication scheme based on the Chuang–Lee’s scheme is proposed that can resist internal attack. In addition, we demonstrate the correctness and security of the improved scheme and analyze its computational costs; (2) to preserve the identity privacy of drivers, anonymity is achieved by randomizing the real identities; and (3) to preserve the location privacy of drivers, unlinkability is achieved in the authentication procedure.

The remainder of this paper is organized as follows. Related work is introduced in Section 2. Preliminaries are presented in Section 3. A review of the Chuang–Lee’s scheme is provided in Section 4. Then, a concrete description of the proposed scheme is offered in Section 5. Section 6 presents the proofs of correctness, security and performance. Finally, the conclusions are provided.

## 2. Related Work

To cope with the challenges associate with VANETs, many types of authentication schemes have been investigated. Porambage et al. [6] introduced a two-phase authentication protocol for sensor networks that uses certificates and consequently cannot preserve the unlinkability of messages. Raya and Hubaux [7] proposed an authentication scheme for VANETs using anonymous certificates, in which each vehicle can utilize distinct key pairs in each authentication stage to avoid being tracked. However, frequent changing of key pairs is likely to result in burdensome management and storage requirements. Lu et al. [8] proposed an alternative way to avoid the complexity of preloading a large number of anonymous certificates with the support of RSUs. When a vehicle passes an RSU, it will be issued a short-term anonymous certificate; thus, the unlinkability of messages is preserved. However, the efficiency will inevitably be low because each vehicle must frequently interact with RSUs. Subsequently, Lin et al. [9] introduced another secure scheme that does not require interaction with RSUs, in which membership managers, rather than RSUs, are responsible for the issuing of certificates based on group signatures. However, the efficiency of this solutions is low. Zhang et al. [10] presented two additional authentication schemes with privacy preservation; however, the computational costs of their methods are somewhat high because of the utilization of bilinear pairing. Similarly, Zheng et al. [11] introduced an authenticated key agreement scheme based on bilinear pairing. Ou et al. [12] later showed that Zheng et al.’s scheme is susceptible to impersonation attacks, and proposed a more secure authenticated key agreement scheme; however, the computational cost of this scheme is again somewhat high because of the utilization of bilinear pairing. In addition, an authentication scheme with access control for VANETs was investigated by Yeh et al. [13]; however, Horng et al. [14] later showed that Yeh et al.’s scheme [13] is susceptible to privilege escalation attacks.

Recently, Chuang and Lee [15] developed a trust-extended authentication mechanism, called TEAM, for VANETs. In TEAM, vehicles are classified into three types, namely, law executors (LEs), mistrusted vehicles (MVs) and trusted vehicles (TVs), as shown in Figure 1. Moreover, it required each vehicle is equipped with a tamper-proof device from which no attacker can extract any stored data, which is so strong that it is not practical. The performance of this mechanism in response to several types of attacks has been analyzed; however, the linkability of messages in the authentication procedure and the possibility of internal attacks during the secure communication procedure, which can easily be executed by a malicious vehicle, have been ignored. A malicious vehicle can trace a driver by intercepting the message sent during the authentication procedure because the values $D_{i}$ and $M_{4}$ are constant. Moreover, a malicious trusted vehicle can compute the real identity of a user and the session key by intercepting a message communicated via the secure communication procedure because it possesses the authorized parameter. Kumari et al. [16] proposed an enhanced trust-extended authentication scheme based on TEAM. However, their scheme fails to protect against internal attacks. Therefore, we have developed an improved authentication procedure and secure communication procedure and have proven their correctness and security. The updating of the constant values used in the authentication procedure is performed by the user himself. Finally, we analyze the computational costs and security features of the improved secure communication procedure.

## 3. Preliminaries

### 3.1. Security Model

To accurately capture the capabilities of an attacker, an experiment concerning the interaction between an adversary and a challenger is introduced. The random oracle model, which originates from the work of Bellare et al. [17], is adopted in our security proof. An adversary *A* can be allowed to communicate with the participants through defined oracle queries; thus, the adversary’s behavior during a real attack can be modeled. In our proposed protocol, each participant is either a common vehicle’s OBU $V_{i}$ or an LE $E_{i}$. Let *U* represent all participants that is the union of common vehicle’s OBUs and LEs.

#### 3.1.1. Protocol Execution

Let $U_{i}^{i}$ represent the *i*th instance of a participant $U_{i}$ and let *b* denote a randomly chosen bit. All possible oracle queries are described as follows:

- $Execute(V_{i}^{i},U_{j}^{i})$: The passive attack capability of the adversary *A* is tested by this query. Executing this query will output an honest execution transcript of the protocol.
- $Send(U_{i}^{i},M)$: The active attack capability of the adversary *A* is tested by this query. *A* can send a Send request on a message *M* to $U_{i}^{i}$. Upon receiving this message, $U_{i}^{i}$ proceeds with the normal execution of the protocol, and then returns the calculated result to the adversary *A*.
- $Corrupt(V_{i}^{i})$: This query models an attack that steals a vehicle’s OBU attack. Upon execution of this query, all the information stored in the OBU of vehicle $U_{i}^{i}$ will be extracted by *A*.
- $Reveal(U_{i}^{i})$: This query models a known key attack. If a session key has been obtained by $U_{i}^{i}$, then the session key of instance $U_{i}^{i}$ is returned to *A*. Otherwise, ⊥ is returned.
- $Test(U_{i}^{i}))$: This query models the ability of the adversary *A* to distinguishing a real session key from a random key. If the session key of participant $U_{i}^{i}$ has not been defined, ⊥ will be returned. Otherwise, if b=1, then the session key of instance $U_{i}^{i}$ will be returned; if b=0, a random key of the same size will be returned.

#### 3.1.2. Notation

An instance $U_{i}^{i}$ is said to have been opened if *A* has issued a query $Reveal(U_{i}^{i})$ to it; otherwise, it is said to be unopened [18]. After receiving the last expected protocol message, $U_{i}^{i}$ enters an accept mode and it is said to be accepted.

#### 3.1.3. Partnering

To illustrate the process of partnering, the concept of a session identification code sid is introduced. Given $U_{1},U_{2}\in OBU$, instances $U_{1}^{i}$ and $U_{2}^{i}$ are called partners only when the following conditions hold: (1) $U_{1}^{i}$ and $U_{2}^{i}$ have entered accept mode. (2) The same sid is shared between $U_{1}^{i}$ and $U_{2}^{i}$. (3) $U_{1}^{i}$ and $U_{2}^{i}$ are partners of each other.

#### 3.1.4. Freshness

To avoid cases in which the security of the scheme is trivially broken by the adversary, the concept of freshness is introduced. The objective is to only permit the adversary to issue Test queries to fresh oracle instances. Specifically, an instance $U_{i}^{i}$ is called fresh when it enters accept mode and both $U_{i}^{i}$ and its partner are unopened.

#### 3.1.5. Semantic Security

Suppose that an adversary *A* executes a protocol *P*. *A* can ask a Test query to a fresh instance after being given access to Execute, Send, Reveal, Corrupt and Test queries, and outputs a guess bit $b^{\prime }$. If $b^{\prime }=b$ where *b* is chosen in the Test query, *A* is said to win this experiment defining semantic security. Let Succ represent the event in which *A* is successful. The advantage of *A* in breaking the semantic security of *P* is defined as follows

$${Adv}_{P,D}\left(A\right)=2Pr\left[Succ\right]-1,$$

where the password is selected from a dictionary *D*.

### 3.2. Elliptic Curve Discrete Logarithm Problem

Let G be an elliptic curve group defined by a generator *P* and a prime number *p*. Then, the two central mathematical problems in elliptic curve cryptography (ECC), namely, the discrete logarithm problem and the computational Diffie–Hellman assumption, can be defined as follows [19].

**Definition** **1.** Elliptic curve discrete logarithm (ECDL) problem. Let Q=aP, where Q,P∈G and $a\in_{R}Z_{p}^{*}$. The objective of the elliptic curve discrete logarithm problem is to find a when given two points Q,P∈G.

**Definition** **2.** *Elliptic curve computational Diffie–Hellman (ECCDH) assumption. Let G denote a representative group of order p and A denote an adversary. Consider the following experiment:*

$$\begin{array}{c} ExperimentExp_{G}^{ECCDH}\left(G,P,p\right),\\ Q_{1}=r_{1}P,Q_{2}=r_{2}P,r_{1},r_{2}\in_{R}Z_{p}^{*},\\ Q=A_{ECCDH}\left(Q_{1},Q_{2}\right),\\ ifQ=r_{1}\cdot r_{2}\cdot P,b=1,else\;b=0,\\ return\;b. \end{array}$$

*The advantage of A in solving the ECCDH problem is defined as follows:*

$$Adv_{G}^{ECCDH}\left(A\right)=\mathrm{Pr}\left[Exp_{G}^{ECCDH}\left(G,P,p\right)=1\right],$$

$$Adv_{G}^{ECCDH}\left(t\right)=\max\left\{Adv_{G}^{ECCDH}\left(A\right)\right\},$$

*where the maximum is taken over all A with time-complexity at most t.*

## 4. Review of the Chuang–Lee’s Scheme

In this section, we review Chuang and Lee’s trust-extended authentication scheme (TEAM) [15]. In their scheme, the vehicles are classified into three types, namely, law executors (LEs), mistrusted vehicles (MVs) and trusted vehicles (TVs), as shown in Figure 1. An LE, such as a police vehicle, is treated as permanently trusted and plays a role similar to that of a mobile authentication server (AS). When a normal vehicle is authenticated successfully, it is deemed to be trusted, otherwise, it is treated as mistrusted. A TV will turn into an MV once the lifetime of its key has expired. To ensure the security of communication, an OBU can obtain service from providers only if it has been authenticated successfully.

TEAM consists of eight procedures: registration, login, password change, general authentication, trusted-extended authentication, secure communication, key update and key revocation. Before each vehicle joins the network, its OBU performs the registration procedure to register itself with the AS. The login procedure is performed when a vehicle intends to access service from the vehicular ad hoc network. After successfully completing the login procedure, the OBU checks its authentication state. If the vehicle is an MV, it needs to perform either the general authentication procedure or the trust-extended authentication procedure; it will then turn into a TV once it has been authenticated successfully and has obtained an authenticated key. Then, it can play the role of an LE to authenticate other mistrusted OBUs via the trust-extended authentication procedure. Two trusted vehicles can perform the secure communication procedure to interact with each other. A trusted vehicle can choose to perform the key update procedure with an LE when its key is approaching expiration. Otherwise, the state of the TV changes to mistrusted when the lifetime of the key has expired.

The OBU of each vehicle is equipped with secure hardware, including a tamper-proof device (TPD) and an event data recorder (EDR). The TPD hinders an attacker from obtaining information from the OBU. Recording important data, such as public parameters, preloaded secret keys, times, and locations, is the responsibility of the EDR. In addition, each vehicle is synchronized via a GPS device. Finally, each vehicle periodically broadcasts a hello message with its authentication state (mistrusted or trusted). The related notations are briefly defined in Table 1. The details of the TEAM protocol follow.

### 4.1. Registration

**Figure 2:**
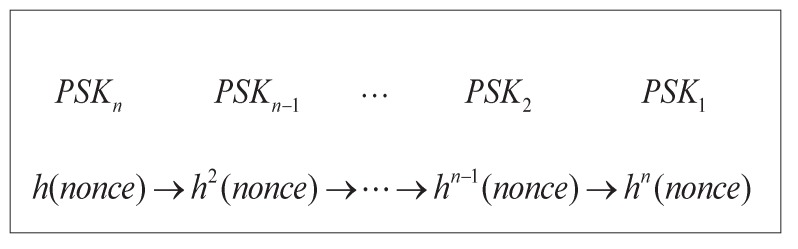
Key set generation scheme based on the hash–chain method.

**LE Registration**: In this procedure, an LE registers itself with the AS via the manufacturer or a secure channel. The secure key set $\left\{PSK_{i},i=1,\ldots ,n\right\}$ is sent to the LE by the AS. Only this secure key set is required to be stored in the secure hardware of the LE. No other user information needs to be stored. Furthermore, the lifetime of each $PSK_{i}$ is set to be short for robust security. When the lifetime of each trusted vehicle’s key expires, this vehicle is required to perform the key update procedure with the LE. The procedure for the key set generation is depicted in Figure 2. It can be seen that the old PSK (e.g., $PSK_{1}$) cannot be used to derive the new PSK (e.g., $PSK_{2}$) because a one-way hash function is introduced in the key generation procedure.

**Normal Vehicle Registration**: All vehicles except LEs need to perform this procedure when they are delivered to market. This registration procedure is performed only once by each vehicle.

Step1. $U_{i}\to AS$: A user $U_{i}$ chooses his password $PW_{i}$ and sends its public identity $ID_{i}$ and $PW_{i}$ to the AS via the manufacturer or a secure channel.
Step2. The AS evaluates the following parameters for $U_{i}$ after it receives $ID_{i}$ and $PW_{i}$: $A_{i}=h\left(ID_{i}∥x\right)$, $B_{i}=h^{2}\left(ID_{i}∥x\right)=h\left(A_{i}\right)$, $C_{i}=h\left(PW_{i}\right)⊕B_{i}$, and $D_{i}=PSK⊕A_{i}$.
Step3. $AS\to U_{i}$: The parameters (i.e., $ID_{i},B_{i},C_{i},D_{i},h\left(\right)$) are stored in the OBU’s secure hardware by the AS via a secure channel.

### 4.2. Login

The login procedure is performed when a user $U_{i}$ intends to access the service from vehicle sensor networks. The login procedure is described as follows:

Step1. $U_{i}\to OBU_{i}$: $ID_{i}$ and $PW_{i}$ are input to $OBU_{i}$ by $U_{i}$.
Step2. First, $OBU_{i}$ verifies $ID_{i}$. Then, it checks whether $B_{i}=h\left(PW_{i}\right)⨁C_{i}$ holds. If so, $OBU_{i}$ launches the general authentication procedure or the trust-extended authentication procedure. Otherwise, the login request will be rejected.

### 4.3. Password Change

When a user $U_{i}$ wants to update his password, he invokes the optional password change procedure. The steps of this procedure are described below:

Step1. $ID_{i}$ and $PW_{i}$ are input to its $OBU_{i}$ by $U_{i}$.
Step2. First, $OBU_{i}$ verifies $ID_{i}$ checks whether $B_{i}=h\left(PW_{i}\right)⊕C_{i}$. If so, $U_{i}$ will be requested to input his new password $PW_{i}^{*}$. $OBU_{i}$ computes $C_{i}^{*}=C_{i}⊕h(PW_{i})⨁h(PW_{i}^{*})$ and replaces $C_{i}$ with $C_{i}^{*}$. Otherwise, the request will be rejected.

### 4.4. General Authentication

The general authentication procedure is performed between $OBU_{i}$ and $LE_{j}$ after $U_{i}$ has completed the login procedure. The steps of this procedure are described below:

Step1. $OBU_{i}$ chooses a random number $r_{i}$ and computes its alias $AID_{i}=h\left(r_{i}\right)⊕ID_{i}$. Then, it produces the request messages $M_{1}=h\left(B_{i}\right)⊕r_{i}$ and $M_{2}=h(r_{i}∥AID_{i}∥D_{i})$.
Step2. $OBU_{i}\to LE_{j}$: The authentication messages (i.e., $AID_{i}$,$M_{1}$,$M_{2}$ and $D_{i}$) are sent from $OBU_{i}$ to $LE_{j}$.
Step3. Upon receiving the authentication request message (i.e., $AID_{i}$,$M_{1}$,$M_{2}$,$D_{i}$), $LE_{j}$ uses PSK to retrieve $A_{i}=D_{i}⊕PSK$ and $r_{i}=M_{1}⊕h^{2}\left(A_{i}\right)$ and then checks whether $M_{2}=h(r_{i}∥AID_{i}∥D_{i})$ holds. The authentication request will be rejected if this equation does not hold. Otherwise, $LE_{j}$ computes $ID_{i}=AID_{i}⊕h(r_{i})$ and produces a random number $r_{j}$ with which to calculate $AID_{j}=r_{j}⊕ID_{j}$ and $SK_{ij}=h(r_{i}∥r_{j})$. Finally, $LE_{j}$ calculates the response messages $M_{3}=r_{j}⊕h^{2}\left(r_{i}\right)$, $M_{4}=A_{i}⊕h(ID_{i})$ and $M_{5}=h(M_{4}∥r_{j}∥AID_{j})$.
Step4. $LE_{j}\to OBU_{i}$: $LE_{j}$ returns its response messages (i.e., $AID_{j}$,$M_{3}$,$M_{4}$,$M_{5}$) to $OBU_{i}$.
Step5. $OBU_{i}$ computes $h^{2}\left(r_{i}\right)$ to retrieve $r_{j}=M_{3}⊕h^{2}\left(r_{i}\right)$ and checks whether $M_{5}=h(M_{4}∥r_{j}∥AID_{j})$ holds. $OBU_{i}$ terminates the process if this equation does not hold. Otherwise, $OBU_{i}$ computes $A_{i}=M_{4}⊕h(ID_{i})$, calculates $SK_{ij}=h(r_{i}∥r_{j})$, and stores $A_{i}$ in its secure hardware.
Step6. $OBU_{i}\to LE_{j}$: The message $SK_{ij}⊕h\left(r_{j}\right)$ is sent to $LE_{j}$ by $OBU_{i}$.
Step7. $LE_{j}$ uses $SK_{ij}$ to retrieve $h(r_{j})$. Then, it checks whether the retrieved hash value is equal to the pre-computed hash value using the chosen $r_{j}$. In this way, a replay attack from an illegal OBU is avoided.

As this time, the state of $OBU_{i}$ changes to trusted since $OBU_{i}$ has been authenticated successfully and has obtained the parameter $PSK=A_{i}⊕D_{i}$. Now, not only LE but also $OBU_{i}$ can authenticate other mistrusted OBUs.

### 4.5. Trust-Extended Authentication

A mistrusted OBU becomes trusted once it has been authenticated successfully and has obtained PSK. Then, it can play the role of an LE to authenticate other mistrusted OBUs. The corresponding trust-extended authentication procedure is the same as the general authentication procedure.

### 4.6. Secure Communication

The secure communication procedure is performed between two trusted vehicles $OBU_{i}$ and $OBU_{j}$ when they intend to interact with each other.

Step1. After completing the login procedure, $OBU_{i}$ generates a random number $r_{i}$ and computes the messages $AID_{i}=ID_{i}⊕r_{i}$, $M_{1}=PSK⊕r_{i}$ and $M_{2}=PSK⊕h(AID_{i}∥r_{i})$, where PSK was obtained in a previous authentication procedure.
Step2. $OBU_{i}\to OBU_{j}$: A secure communication request (i.e., $AID_{i}$,$M_{1}$,$M_{2}$) is sent to $OBU_{j}$ by $OBU_{i}$.
Step3. Upon receiving (i.e., $AID_{i}$,$M_{1}$,$M_{2}$), $OBU_{j}$ uses PSK to retrieve $r_{i}$ from $M_{1}$ and then computes $PSK⊕h(AID_{i}∥r_{i})$ and checks whether it is equal to $M_{2}$. The request will be rejected if this equality does not hold. Otherwise, $OBU_{j}$ randomly chooses $r_{j}$ and computes $AID_{j}=ID_{j}⊕r_{j}$, $M_{3}=PSK⊕r_{j}$, $M_{4}=PSK⊕h(AID_{j}∥r_{j}∥h\left(r_{i}\right))$ and a session key $SK_{ij}=h(r_{i}∥r_{j}∥PSK)$.
Step4. $OBU_{j}\to OBU_{i}$: $OBU_{j}$ returns the response messages (i.e., $AID_{j}$,$M_{3}$,$M_{4}$) o $OBU_{i}$.
Step5. After receiving the messages $\{AID_{j}$,$M_{3}$,$M_{4}\}$, $OBU_{i}$ verifies whether $OBU_{j}$ is trusted: $OBU_{i}$ uses PSK to retrieve $r_{j}$ from $M_{3}$ and checks whether $M_{4}=h(AID_{j}∥r_{j}∥h\left(r_{i}\right))$ holds. If so, $OBU_{i}$ computes a session key $SK_{ij}=h(r_{i}∥r_{j}∥PSK)$ and a reply message $M_{5}=SK_{ij}⊕h\left(r_{j}\right)$. Otherwise, the process is terminated.
Step6. $OBU_{i}\to OBU_{j}$: $OBU_{i}$ sends $\left(M_{5}\right)$ to $OBU_{j}$.
Step7. After receiving the message $M_{5}$, $OBU_{j}$ computes $SK_{ij}⊕h\left(r_{j}\right)$ and then checks whether it is equal to $M_{5}$. If this quality holds, then the two trusted vehicles can communicate securely using $SK_{ij}$. Otherwise, $OBU_{j}$ terminates the process.

### 4.7. Key Revocation

Key revocation will be triggered when the lifetime of a key expires. The state of a mistrusted vehicle changes to trusted when the mistrusted vehicle is authenticated successfully and obtains PSK via performing either the general authentication procedure or the trust-extended authentication procedure. Then, a timer is instantiated by the secure hardware and begins to count down. The state of the vehicle becomes mistrusted when the lifetime of the key expires. When key expiration is approaching, the system requests that the trusted vehicle performs the key update procedure.

### 4.8. Key Update

The key update procedure will be invoked by $OBU_{i}$ when the key lifetime of the TV is approaching expiration. The steps of this procedure are described as follows.

Step1. $OBU_{i}$ randomly chooses $r_{i}$ to compute the messages $M_{1}=PSK_{old}⊕r_{i}$, $M_{2}=PSK_{old}\;MSG_{KU}$, and $M_{3}=h(r_{i}∥MSU_{KU})$.
Step2. $OBU_{i}\to LE_{j}$: A key update request (i.e., $M_{1}$,$M_{2}$,$M_{3}$) is sent to $LE_{j}$ by $OBU_{i}$.
Step3. $LE_{j}$ retrieves $r_{i}$ and $MSG_{KU}$ using the current PSK (i.e., $PSK_{old}$). The key update request will be rejected if $h(r_{i}∥MSG_{KU})$ does not match $M_{3}$. Otherwise, $LE_{j}$ chooses a random number $r_{j}$ and computes $M_{4}=r_{j}⊕h\left(r_{i}\right)$, $M_{5}=PSK_{new}⊕r_{j}$, and $M_{6}=h(r_{j}∥PSK_{new})$, where PSK is produced via the hash–chain method. Therefore, the new PSK cannot be inferred by other OBUs using the current PSK. Finally, $LE_{j}$ computes $SK_{ij}=h(r_{i}∥r_{j}∥PSK_{new})$.
Step4. $LE_{j}\to OBU_{i}$: $LE_{j}$ returns the reply messages (i.e., $M_{4}$,$M_{5}$, and $M_{6}$) to $OBU_{i}$.
Step5. Upon receiving the reply messages, $OBU_{i}$ computes $h(r_{i})$ to retrieve $r_{j}=M_{4}⊕h(r_{i})$, and obtains $PSK_{new}=M_{5}⊕r_{j}$. Next, $OBU_{i}$ checks whether $M_{6}=h(r_{j}∥PSK_{new})$ and $PSK_{old}=h\left(PSK_{new}\right)$. If this condition holds, $OBU_{i}$ renews the PSK and computes $SK_{ij}=h(r_{i}∥r_{j}∥PSK_{new})$. Otherwise, $OBU_{i}$ terminates the process.
Step6. $OBU_{i}\to LE_{j}$: $OBU_{i}$ sends the message $SK_{ij}⊕h(r_{j})$ to $LE_{j}$.
Step7. $LE_{j}$ retrieves $h(r_{j})$ using $SK_{ij}$. Then, it checks whether the retrieved hash value is equal to the pre-computed hash value using the chosen $r_{j}$. In this way, a replay attack from an illegal OBU is avoided. Now, this session key can be used to communicate securely between two trusted vehicles.

## 5. Improved Scheme

A concrete description of our enhanced privacy-preserving authentication scheme is presented in this section. In our scheme, the vehicles are also classified into three types: law executors (LEs), mistrusted vehicles (MVs) and trusted vehicles (TVs) as displayed in Figure 1. The LEs are equipped with TPD, but the normal vehicles such as TV and MV are not equipped with TPD. Our improved scheme consists of nine procedures: initialization, registration, login, password change, general authentication, trust-extended authentication, secure communication, key update and revocation. The notations used in this section are also briefly defined in Table 1.

### 5.1. Initialization

The initialization procedure is performed by the AS when it sets up the system parameters:

Step1. Let G be an elliptic curve group defined by a generator *P* and a prime number *p*. The AS randomly selects $\left\{x\in Z_{p}^{*}\right\}$ as its secret key.
Step2. The AS computes the secure key set $\left\{PSK_{i},i=1,\ldots ,n\right\}$ using the hash–chain method as shown in Figure 2, e.g., $h^{2}\left(x\right)=h\left(h\left(x\right)\right)$.

### 5.2. Registration

**LE Registration**: In this procedure, an LE registers itself with the AS via the manufacturer or a secure channel. The secure key set $\left\{PSK_{i},i=1,\ldots ,n\right\}$ and the public parameters {G,p,P} are sent to the LE by the AS. Only the secure key set and the public parameters are required to be stored in the secure hardware of the LE. No other user information needs to be stored. Similarly, the lifetime of each $PSK_{i}$ is set to be short for robust security. When the lifetime of each trusted vehicle’s key expires, this vehicle is required to perform the key update procedure with an LE.

**Figure 3:**
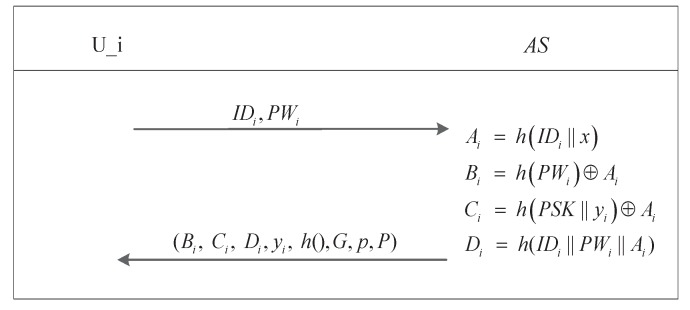
Normal vehicle registration procedure.

**Normal Vehicle Registration**: All vehicles except LEs need to perform this procedure when they are delivered to market. This registration procedure is performed only once by each vehicle. The steps of the normal vehicle registration procedure are described in Figure 3.

Step1. $U_{i}\to AS$: A user $U_{i}$ chooses his password $PW_{i}$ and sends its public identity $ID_{i}$ and $PW_{i}$ to the AS via the manufacturer or a secure channel.
Step2. The AS chooses a random number $y_{i}$ with which to evaluate the following parameters for $U_{i}$ after it receives $ID_{i}$ and $PW_{i}$: $A_{i}=h\left(ID_{i}∥x\right)$, $B_{i}=h\left(PW_{i}\right)⊕A_{i}$,$C_{i}=h\left(PSK∥y_{i}\right)⊕A_{i}$, and $D_{i}=h(ID_{i}∥PW_{i}∥A_{i})$.
Step3. $AS\to U_{i}$: The parameters (i.e., $B_{i}$, $C_{i}$, $D_{i}$, $y_{i}$, h,*G*,*p*,*P*) are stored in the OBU’s secure hardware by the AS via a secure channel.
Step4. $U_{i}$ chooses a number $x_{i}$ as his private key and computes $P_{pub_{i}}=x_{i}P$ as his public key, and then computes $Z_{i}=x_{i}⊕h\left(PW_{i}\right)$ and stores ($P_{pub_{i}}$, $Z_{i}$) in its OBU secure hardware.

### 5.3. Login

The login procedure is performed when a user $U_{i}$ intends to access service from the vehicle sensor network. The login procedure is described as follows:

Step1. $U_{i}\to OBU_{i}$: $ID_{i}$ and $PW_{i}$ are input to $OBU_{i}$ by $U_{i}$.
Step2. First, $OBU_{i}$ retrieves $A_{i}=h\left(PW_{i}\right)⊕B_{i}$. Then, it checks whether $D_{i}=h(ID_{i}∥PW_{i}∥A_{i})$ holds. If so, $OBU_{i}$ launches the general authentication procedure or the trust-extended authentication procedure. Otherwise, the login request will be rejected.

### 5.4. Password Change

When a user $U_{i}$ wants to update his password, the optional password change procedure will be invoked. The steps of this procedure are described as follows:

Step1. $ID_{i}$ and $PW_{i}$ are input to $OBU_{i}$ by $U_{i}$.
Step2. First, $OBU_{i}$ retrieves $A_{i}=h\left(PW_{i}\right)⊕B_{i}$. Then, it checks whether $D_{i}=h(ID_{i}∥PW_{i}∥A_{i})$ holds. If so, $U_{i}$ will be requested to input his new password $PW_{i}^{*}$. $OBU_{i}$ computes $B_{i}^{*}=B_{i}⊕h(PW_{i})⨁h(PW_{i}^{*})$ and $D_{i}^{*}=h(ID_{i}∥PW_{i}^{*}∥A_{i})$ and replaces $B_{i}$ and $D_{i}$ with $B_{i}^{*}$ and $D_{i}^{*}$. Otherwise, the request will be rejected.

### 5.5. General Authentication

**Figure 4:**
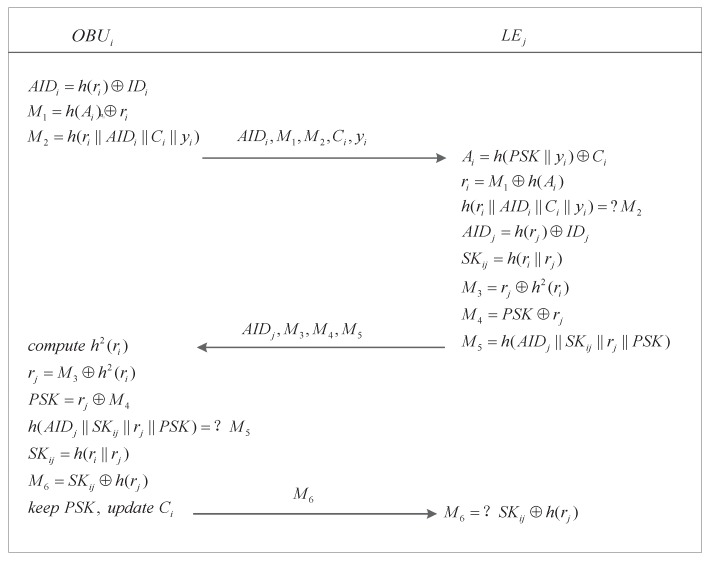
General authentication procedure.

The general authentication procedure is performed between $OBU_{i}$ and $LE_{j}$ after $U_{i}$ has completed the login procedure. The general authentication procedure is shown in Figure 4 and the steps are described as follows.

Step1. $OBU_{i}$ chooses a random number $r_{i}$ and computes its alias $AID_{i}=h\left(r_{i}\right)⊕ID_{i}$. Then, it produces the request messages $M_{1}=h\left(A_{i}\right)⊕r_{i}$ and $M_{2}=h(r_{i}∥AID_{i}∥C_{i}∥y_{i})$, where $A_{i}$ is obtained from the login procedure.
Step2. $OBU_{i}\to LE_{j}$: The authentication messages (i.e., $AID_{i}$, $M_{1}$, $M_{2}$, $C_{i}$, and $y_{i}$) are sent from $OBU_{i}$ to $LE_{j}$.
Step3. Upon receiving the authentication request messages (i.e., $AID_{i}$, $M_{1}$, $M_{2}$, $C_{i}$, and $y_{i}$), $LE_{j}$ uses PSK to retrieve $A_{i}=C_{i}⊕h(PSK∥y_{i})$ and $r_{i}=M_{1}⊕h(A_{i})$ and then checks whether $M_{2}=h(r_{i}∥AID_{i}∥C_{i}∥y_{i})$ holds. The authentication request will be rejected if it does not. Otherwise, $LE_{j}$ produces a random number $r_{j}$ to calculate $AID_{j}=ID_{j}⊕h\left(r_{j}\right)$ and $SK_{ij}=h(r_{i}∥r_{j})$. Finally, $LE_{j}$ calculates the response messages $M_{3}=r_{j}⊕h^{2}\left(r_{i}\right)$, $M_{4}=PSK⊕r_{j}$, and $M_{5}=h(AID_{j}∥SK_{ij}∥r_{j}∥PSK)$.
Step4. $LE_{j}\to OBU_{i}$: $LE_{j}$ return response messages (i.e., $AID_{j}$, $M_{3}$, $M_{4}$, and $M_{5}$) to $OBU_{i}$.
Step5. $OBU_{i}$ computes $h^{2}\left(r_{i}\right)$ to retrieve $r_{j}=M_{3}⊕h^{2}\left(r_{i}\right)$, $PSK=M_{4}⊕r_{j}$, and $SK_{ij}=h(r_{i}∥r_{j})$ and checks whether $M_{5}=h(AID_{j}∥SK_{ij}∥r_{j}∥PSK)$ holds. $OBU_{i}$ terminates the process if it does not. Otherwise, $OBU_{i}$ calculates the reply message $M_{6}=SK_{ij}⊕h\left(r_{j}\right)$; computes $C_{inew}=h(PSK∥r_{i})⊕A_{i}$ and $E_{i}=h\left(PW_{i}\right)⊕PSK$; replaces $C_{i}$ and $y_{i}$ with $C_{inew}$ and $r_{i}$, respectively, and stores $E_{i}$ in its secure hardware.
Step6. $OBU_{i}\to LE_{j}$: The message $M_{6}$ is sent to to $LE_{j}$ by $OBU_{i}$.
Step7. $LE_{j}$ uses $SK_{ij}$ to retrieve $h(r_{j})$. Then, it checks whether the retrieved hash value is equal to the pre-computed hash value using the chosen $r_{j}$. In this way, a replay attack from an illegal OBU is avoided .

At this time, the state of $OBU_{i}$ changes to trusted since $OBU_{i}$ has been authenticated successfully and has obtained the parameter PSK. Now, not only LE but also $OBU_{i}$ can authenticate other mistrusted OBUs.

### 5.6. Trust-Extended Authentication

This procedure is the same as in the Chuang–Lee scheme.

### 5.7. Secure Communication

**Figure 5:**
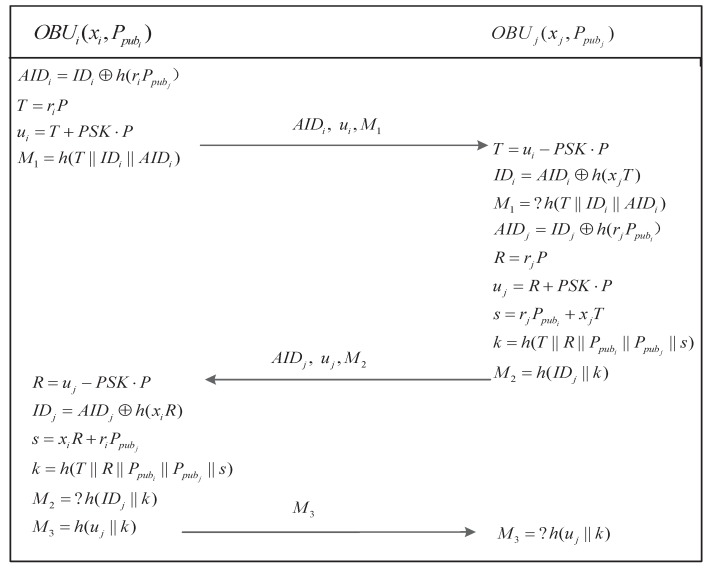
Secure communication procedure.

The secure communication procedure is performed between two trusted vehicles $OBU_{i}$ and $OBU_{j}$ when they intend to interact with each other. The secure communication procedure is shown in Figure 5 and the steps are described as follows.

Step1. After completing the login procedure, $OBU_{i}$ retrieves $PSK=E_{i}⊕h\left(PW_{i}\right)$,$x_{i}=Z_{i}⊕h\left(PW_{i}\right)$, then it generates a random number $r_{i}$ and computes the messages $AID_{i}=ID_{i}⊕h\left(r_{i}P_{{pub}_{j}}\right)$, $T=r_{i}P$, $u_{i}=T+PSK\cdot P$, and $M_{1}=h(T∥ID_{i}∥AID_{i})$, where $E_{i}$ was obtained from a previous authentication procedure.
Step2. $OBU_{i}\to OBU_{j}$: A secure communication request (i.e., $AID_{i}$, $u_{i}$, $M_{1}$) is sent to $OBU_{j}$ by $OBU_{i}$.
Step3. Upon receiving (i.e., $AID_{i}$, $u_{i}$, $M_{1}$), $OBU_{j}$ uses PSK to retrieve *T* from $u_{i}$ and then computes $ID_{i}=AID_{i}⊕h\left(x_{j}T\right)$, and checks whether $M_{1}$ is equal to $h(T∥ID_{i}∥AID_{i})$. The request will be rejected if this equality does not holds. Otherwise, $OBU_{j}$ randomly chooses $r_{j}$ and computes
  $$\begin{array}{c} AID_{j}=ID_{j}⊕h\left(r_{j}P_{{pub}_{i}}\right),\\ R=r_{j}P,\\ u_{j}=R+PSK\cdot P,\\ s=r_{j}P_{{pub}_{i}}+x_{j}T,\\ k=h(T∥R∥P_{{pub}_{i}}∥P_{{pub}_{j}}∥s),\\ M_{2}=h(ID_{j}∥k). \end{array}$$
Step4. $OBU_{j}\to OBU_{i}$: $OBU_{j}$ returns the response messages (i.e., $AID_{j}$, $u_{j}$, $M_{2}$) to $OBU_{i}$.
Step5. After receiving the messages $\left\{AID_{j},u_{j},M_{2}\right\}$, $OBU_{i}$ verifies whether $OBU_{j}$ is trusted: $OBU_{i}$ computes $R=u_{j}-PSK\cdot P$, $ID_{j}=AID_{j}⊕h\left(x_{i}R\right)$, $s=r_{i}P_{{pub}_{j}}+x_{i}R$ and $k\;=\;h(T∥R∥P_{{pub}_{i}}∥P_{{pub}_{j}}∥s)$, and then checks whether $M_{2}=h(ID_{j}∥k)$ holds. If so, $OBU_{i}$ computes a reply message $M_{3}=h(u_{j}∥k)$. Otherwise, the process is terminated.
Step6. $OBU_{i}\to OBU_{j}$: $OBU_{i}$ sends $M_{3}$ to $OBU_{j}$.
Step7. After receiving the message $\left\{M_{3}\right\}$, $OBU_{j}$ checks whether $M_{3}=h(u_{j}∥k)$ holds. if so, the two trusted vehicles can communicate securely using *k*. Otherwise, $OBU_{j}$ terminates the process.

### 5.8. Key Revocation

This procedure is the same as in the Chuang–Lee scheme.

### 5.9. Key Update

This procedure is the same as in the Chuang–Lee scheme.

## 6. Analysis

In this section, we first validate the correctness of the critical general authentication procedure and secure communication procedure using the BAN logic, and we then prove the security of our improved scheme. Finally, we evaluate the performance of our scheme against that of the existing related schemes.

### 6.1. Correctness

The BAN logic is a useful way to validate the correctness of security protocols, especially for the authentication protocols [20]. Some relevant notations are listed in Table 2. The verification procedure consists of the following steps.

#### 6.1.1. The Correctness of the General Authentication Procedure

##### Idealization

First, we use formal logical language to idealize the general authentication procedure in our improved scheme in accordance with the rules of the BAN logic as follows:

(1). $OBU_{i}\to LE_{j}$ : $\{M_{1}=h(r_{i}∥AID_{i}∥C_{i}∥y_{i}),AID_{i},{\left\{r_{i}\right\}}_{A_{i}},{\left\{A_{i}\right\}}_{PSK}\},$
(2). $LE_{j}\to OBU_{i}$ : $\{M_{2}=h(AID_{j}∥SK_{ij}∥r_{j}∥PSK),AID_{j},{\left\{r_{j}\right\}}_{r_{i}},{\left\{PSK\right\}}_{r_{j}}\},$
(3). $OBU_{i}\to LE_{j}$ : $\{M_{3}=SK_{ij}⊕h\left(r_{j}\right).$

##### Goal

There are two roles in the general authentication procedure: $OBU_{i}$ and $LE_{j}$. Since $OBU_{i}$ needs to obtain the authorized parameter PSK from the $LE_{j}$, it must believe PSK. Moreover, $OBU_{i}$ and $LE_{j}$ must believe each other and each other’s aliases, and they must believe the session key computed in the general authentication procedure. Thus, there are five goals of the general authentication procedure in our improved scheme as follows:

G1. $OBU_{i}|\equiv LE_{j}|\equiv PSK$: $OBU_{i}$ believes PSK.
G2. $OBU_{i}|\equiv LE_{j}|\equiv AID_{j}$: $OBU_{i}$ believes $LE_{j}$ and his alias $AID_{j}$.
G3. $LE_{j}|\equiv OBU_{i}|\equiv AID_{i}$: $LE_{j}$ believes $OBU_{i}$ and his alias $AID_{i}$.
G4. $OBU_{i}|\equiv OBU_{i}\overset{SK_{ij}}{⟷}LE_{j}$: $OBU_{i}$ believes the share key between himself and $LE_{j}$.
G5. $LE_{j}|\equiv$$LE_{j}\overset{SK_{ij}}{⟷}OBU_{i}$: $LE_{j}$ believes the share key between himself and $OBU_{i}$.

##### Assumptions

With the goals set, the assumptions also need to be stated as follows:

A1. $OBU_{i}◃AID_{i}$: $OBU_{i}$ possesses an alias $AID_{i}$.
A2. $LE_{j}◃AID_{j}$: $OBU_{j}$ possesses an alias $AID_{j}$.
A3. $OBU_{i}|\equiv ♯\left(r_{i},r_{j}\right)$: $OBU_{i}$ believes the freshness of $r_{i}$ and $r_{j}$.
A4. $LE_{j}|\equiv ♯\left(r_{i},r_{j},y_{i}\right)$: $LE_{j}$ believes the freshness of $r_{i}$, $r_{j}$ and $y_{i}$.
A5. $LE_{j}|\equiv$$LE_{j}\overset{PSK}{⟷}OBU_{i}$: $LE_{j}$ believes the share key PSK between himself and $OBU_{i}$.
A6. $OBU_{i}|\equiv$$OBU_{i}\overset{r_{i}}{⟷}LE_{j}$: $OBU_{i}$ believes the share key $r_{i}$ between himself and $LE_{j}$.
A7. $LE_{j}|\equiv$$LE_{j}\overset{r_{j}}{⟷}OBU_{i}$: $LE_{j}$ believes the share key $r_{j}$ between himself and $OBU_{i}$.

##### Verification

In this subsection, we will verify the correctness of our proposed general authentication procedure using the BAN logic. The detailed steps of the proof are as follows:

$OBU_{i}$ computes $AID_{i}$ and ${\left\{r_{i}\right\}}_{A_{i}}:$

V1. $\frac{LE_{j}⊲{\left\{r_{i}\right\}}_{A_{i}},AID_{i},{\left\{A_{i}\right\}}_{PSK},M_{2},y_{i},LE_{j}|\equiv LE_{j}\overset{PSK}{⟷}OBU_{i}}{OBU_{j}|\equiv OBU_{i}∼M_{2}},$
V2. $\frac{LE_{j}|\equiv ♯\left(r_{i},y_{i}\right),LE_{j}|\equiv OBU_{i}∼M_{2}}{LE_{i}|\equiv OBU_{i}|\equiv M_{2}}$,
V3. $\frac{LE_{j}|\equiv OBU_{i}|\equiv M_{2}}{LE_{j}|\equiv OBU_{i}|\equiv AID_{i}},$

$LE_{j}$ computes $LE_{j}\overset{SK_{ij}}{⟷}OBU_{i}$, ${\left\{r_{j}\right\}}_{r_{i}}$, ${\left\{PSK\right\}}_{r_{j}},$

V4. $\frac{OBU_{i}⊲AID_{j},{\left\{r_{j}\right\}}_{r_{i}},{\left\{PSK\right\}}_{r_{j}},M_{5},OBU_{i}|\equiv OBU_{i}\overset{r_{i}}{⟷}LE_{j}}{OBU_{i}|\equiv LE_{j}∼M_{5}}$,
V5. $\frac{OBU_{i}|\equiv ♯\left(r_{i},r_{j}\right),OBU_{i}|\equiv LE_{j}∼M_{5}}{OBU_{i}|\equiv LE_{j}|\equiv M_{5}}$,
V6. $\frac{OBU_{i}|\equiv LE_{j}|\equiv M_{5}}{OBU_{i}|\equiv LE_{j}|\equiv r_{j}}$,
V7. $\frac{OBU_{i}|\equiv ♯\left(r_{i},r_{j}\right)}{OBU_{i}|\equiv ♯\left(SK_{ij}\right)}$,
V8. $\frac{OBU_{i}|\equiv ♯\left(SK_{ij}\right),OBU_{i}|\equiv LE_{j}|\equiv r_{j}}{OBU_{i}|\equiv OBU_{i}\overset{SK_{ij}}{⟷}LE_{j}}$,
V9. $\frac{OBU_{i}|\equiv LE_{j}|\equiv M_{5}}{OBU_{i}|\equiv LE_{j}|\equiv AID_{j}}$,
V10. $\frac{OBU_{i}|\equiv LE_{j}|\equiv M_{5}}{OBU_{i}|\equiv LE_{j}|\equiv PSK},$

$OBU_{i}$ computes $M_{6},$

V11. $\frac{LE_{j}⊲SK_{ij},M_{6},LE_{j}|\equiv LE_{j}\overset{r_{j}}{⟷}OBU_{i}}{LE_{j}|\equiv OBU_{i}∼M_{6}}$,
V12. $\frac{LE_{j}|\equiv ♯\left(r_{j}\right),LE_{j}|\equiv OBU_{i}∼M_{6}}{LE_{j}|\equiv OBU_{i}|\equiv M_{6}}$,
V13. $\frac{LE_{j}|\equiv OBU_{i}|\equiv M_{6}}{LE_{j}|\equiv OBU_{i}|\equiv r_{i}}$,
V14. $\frac{LE_{j}|\equiv ♯\left(r_{i},r_{j}\right)}{OBU_{j}|\equiv ♯SK_{ij}}$,
V15. $\frac{LE_{j}|\equiv ♯\left(SK_{ij}\right),LE_{j}|\equiv OBU_{i}|\equiv r_{i}}{LE_{j}|\equiv LE_{j}\overset{SK_{ij}}{⟷}OBU_{i}}.$

In formula V3 and formulas V9 and V10, $LE_{j}$ believes that $OBU_{i}$ has sent $M_{2}$ and $OBU_{i}$ believes that $LE_{j}$ has sent $M_{5}$. Because $LE_{j}$ has verified the correctness of message $M_{2}$ and $OBU_{i}$ has verified the correctness of message $M_{5}$, $OBU_{i}$ and $LE_{j}$ each believe the other party and its alias, and $OBU_{i}$ believes the PSK obtained from $LE_{j}$. In formula V8, because $OBU_{i}$ is able to calculate $r_{j}$ and believes this value which is necessary to compute $SK_{ij}$, $OBU_{i}$ believes the freshness of $SK_{ij}$, and $OBU_{i}$ believes the session key $SK_{ij}$ that it computes. Similarly, in formula V15, $LE_{j}$ believes the value $r_{i}$ and the freshness of $SK_{ij}$, thus $OBU_{i}$ believes the session key $SK_{ij}$ that it computes. According to formulas V3, V8, V9, V10 and V15, we can infer that our improved general authentication procedure achieves our goals.

#### 6.1.2. The Correctness of the Secure Communication Procedure

##### Idealization

First, we use formal logical language to idealize the secure communication procedure in our improved scheme in accordance with the rules of the BAN logic as follows:

(1). $OBU_{i}\to OBU_{j}$ : $\{M_{1}=h(r_{i}P∥ID_{i}∥{\left\{ID_{i}\right\}}_{P_{pub_{j}}}),{\left\{ID_{i}\right\}}_{P_{pub_{j}}},{\left\{r_{i}P\right\}}_{PSK}\},$
(2). $OBU_{j}\to OBU_{i}$ : $\{M_{2}=h\left(ID_{j}∥k\right),{\left\{ID_{j}\right\}}_{P_{pub_{i}}},{\left\{r_{j}P\right\}}_{PSK}\},$
(3). $OBU_{i}\to OBU_{j}$ : $\{M_{3}=h\left({\left\{r_{j}P\right\}}_{PSK}∥k\right)\}.$

##### Goal

There are two roles in the secure communication procedure: $OBU_{i}$ and $OBU_{j}$, which are the on-board units of the two communication vehicles. Since $OBU_{i}$ and $OBU_{j}$ need to generate a common session key for their communication, they must believe each other and each other’s identities, and they must believe the session key computed in the secure communication procedure. Thus, there are four goals of the secure communication procedure in our improved scheme as follows:

G1. $OBU_{i}|\equiv OBU_{j}|\equiv ID_{j}$: $OBU_{i}$ believes $OBU_{j}$ and its identity $ID_{j}$.
G2. $OBU_{j}|\equiv OBU_{i}|\equiv ID_{i}$: $OBU_{j}$ believes $OBU_{i}$ and its identity $ID_{i}$.
G3. $OBU_{i}|\equiv OBU_{i}\overset{k}{⟷}OBU_{j}$: $OBU_{i}$ believes the shared key between itself and $OBU_{j}$.
G4. $OBU_{j}|\equiv$$OBU_{j}\overset{k}{⟷}OBU_{i}$: $OBU_{j}$ believes the shared key between itself and $OBU_{i}$.

##### Assumptions

With the goals set, the assumptions also need to be stated as follows:

A1. $OBU_{i}◃ID_{i}$: $OBU_{i}$ owns its identity $ID_{i}$.
A2. $OBU_{j}◃ID_{j}$: $OBU_{j}$ owns its identity $ID_{j}$.
A3. $OBU_{i}◃x_{i}$: $OBU_{i}$ holds own private key $x_{i}$.
A4. $OBU_{j}◃x_{j}$: $OBU_{j}$ holds own private key $x_{j}$.
A5. $OBU_{i}|\equiv \overset{P_{pub_{i}}}{\mapsto }OBU_{i}$: $OBU_{i}$ believes own public key $P_{pub_{i}}$.
A6. $OBU_{j}|\equiv \overset{P_{pub_{j}}}{\mapsto }OBU_{j}$: $OBU_{j}$ believes own public key $P_{pub_{j}}$.
A7. $OBU_{i}◃\left(P_{pub_{i}},P_{pub_{j}}\right)$: $OBU_{i}$ holds own public key $P_{pub_{i}}$ and $OBU_{j}$’s public key $P_{pub_{j}}$.
A8. $OBU_{j}◃\left(P_{pub_{i}},P_{pub_{j}}\right)$: $OBU_{j}$ holds own public key $P_{pub_{j}}$ and $OBU_{i}$’s public key $P_{pub_{i}}$.
A9. $OBU_{i}|\equiv ♯\left(r_{i},r_{j}\right)$: $OBU_{i}$ believes the freshness of $r_{i}$ and $r_{j}$.
A10. $OBU_{j}|\equiv ♯\left(r_{i},r_{j}\right)$: $OBU_{j}$ believes the freshness of $r_{i}$ and $r_{j}$.
A11. $OBU_{i}|\equiv$$OBU_{i}\overset{PSK}{⟷}OBU_{j}$: $OBU_{i}$ believes the share key PSK between himself and $OBU_{j}$.
A12. $OBU_{j}|\equiv$$OBU_{j}\overset{PSK}{⟷}OBU_{i}$: $OBU_{j}$ believes the share key PSK between himself and $OBU_{i}$.

##### Verification

In this subsection, we will verify the correctness of our proposed secure communication procedure using the BAN logic. The detailed steps of the proof are as follows:

$OBU_{i}$ computes ${\left\{ID_{i}\right\}}_{P_{pub_{j}}}$ and ${\left\{r_{i}P\right\}}_{PSK}$

V1. $\frac{OBU_{j}|\equiv \overset{P_{pub_{j}}}{\mapsto }OBU_{j},OBU_{j}⊲{\left\{ID_{i}\right\}}_{P_{pub_{j}}}}{OBU_{j}⊲ID_{i}}$,
V2. $\frac{OBU_{j}⊲{\left\{r_{i}P\right\}}_{PSK},ID_{i},M_{1},OBU_{j}|\equiv OBU_{j}\overset{PSK}{⟷}OBU_{i}}{OBU_{j}|\equiv OBU_{i}∼M_{1}}$,
V3. $\frac{OBU_{j}|\equiv ♯\left(r_{i}\right),OBU_{j}|\equiv OBU_{i}∼M_{1}}{OBU_{j}|\equiv OBU_{i}|\equiv M_{1}}$,
V4. $\frac{OBU_{j}|\equiv OBU_{i}|\equiv M_{1}}{OBU_{j}|\equiv OBU_{i}|\equiv ID_{i}}.$

$OBU_{j}$ computes $OBU_{j}\overset{k}{⟷}OBU_{i}$ , ${\left\{r_{j}P\right\}}_{PSK}$

V5. $\frac{OBU_{i}|\equiv \overset{P_{pub_{i}}}{\mapsto }OBU_{i},OBU_{j}⊲{\left\{ID_{j}\right\}}_{P_{pub_{i}}}}{OBU_{i}⊲ID_{j}}$,
V6. $\frac{OBU_{i}⊲{\left\{r_{j}P\right\}}_{PSK},r_{i},x_{i},P_{pub_{i}},P_{pub_{j}},M_{2},OBU_{i}|\equiv OBU_{i}\overset{PSK}{⟷}OBU_{j}}{OBU_{i}|\equiv OBU_{j}∼M_{2}}$,
V7. $\frac{OBU_{i}|\equiv ♯\left(r_{i},r_{j}\right),OBU_{i}|\equiv OBU_{j}∼M_{2}}{OBU_{i}|\equiv OBU_{j}|\equiv M_{2}}$,
V8. $\frac{OBU_{i}|\equiv OBU_{j}|\equiv M_{2}}{OBU_{i}|\equiv OBU_{j}|\equiv ID_{j}}$,
V9. $\frac{OBU_{i}|\equiv OBU_{j}|\equiv M_{2}}{OBU_{i}|\equiv OBU_{j}|\equiv s},$

V10. $\frac{OBU_{i}|\equiv ♯\left(r_{i},r_{j}\right)}{OBU_{i}|\equiv ♯\left(k\right)}$,
V11. $\frac{OBU_{i}|\equiv ♯\left(k\right),OBU_{i}|\equiv OBU_{j}|\equiv s}{OBU_{i}|\equiv OBU_{i}\overset{k}{⟷}OBU_{j}}.$

$OBU_{i}$ computes $M_{3}$

V12. $\frac{OBU_{j}⊲{\left\{r_{i}P\right\}}_{PSK},k,M_{3},OBU_{j}|\equiv OBU_{j}\overset{PSK}{⟷}OBU_{i}}{OBU_{j}|\equiv OBU_{i}∼M_{3}},$
V13. $\frac{OBU_{j}|\equiv ♯\left(r_{i},r_{j}\right),OBU_{j}|\equiv OBU_{i}∼M_{3}}{OBU_{j}|\equiv OBU_{i}|\equiv M_{3}},$
V14. $\frac{OBU_{j}|\equiv OBU_{i}|\equiv M_{3}}{OBU_{j}|\equiv OBU_{i}|\equiv s},$
V15. $\frac{OBU_{j}|\equiv ♯\left(r_{i},r_{j}\right)}{OBU_{j}|\equiv ♯\left(k\right)}$,
V16. $\frac{OBU_{j}|\equiv ♯\left(k\right),OBU_{j}|\equiv OBU_{i}|\equiv s}{OBU_{j}|\equiv OBU_{j}\overset{k}{⟷}OBU_{i}}$.

In formula V4 and formula V8, $OBU_{j}$ believes that $OBU_{i}$ has sent $M_{1}$ and $OBU_{i}$ believes that $OBU_{j}$ has sent $M_{2}$. Because $OBU_{j}$ has verified the correctness of message $M_{1}$ and $OBU_{i}$ has verified the correctness of message $M_{2}$ , $OBU_{i}$ and $OBU_{j}$ each believe the other’s identity and that the other party is a trusted vehicle. In formula V11, because $OBU_{i}$ can use its private key to obtain $ID_{j}$ and calculate *k*, $OBU_{i}$ can verify $M_{2}$ by means of $ID_{j}$ and *k*; thus, $OBU_{i}$ believes the session key *k* that it computes. Similarly, in formula V16, $OBU_{j}$ can compute the session key *k* to verify $M_{3}$, so $OBU_{j}$ believes the session key *k* that it computes. According to formulas V4, V8, V14 and V16, we can infer that our improved secure communication procedure achieves our goals.

### 6.2. Security Analysis

In this section, the security proof of the critical secure communication procedure and general authentication procedure is presented. We show that the proposed improved protocol is secure through a formal security analysis in the random oracle model as well as an informal security analysis.

#### 6.2.1. The Formal Security Analysis

**Theorem** **1.** *Let GAP denote the general authentication procedure presented in Figure 4. Let |Hash| and |D| denote the range space of the hash function and the size of the password dictionary D, respectively. Finally, let A represent an adversary within a polynomial time t against the semantic security of GAP by issuing $q_{send}$ Send queries, $q_{exe}$ Execute queries and $q_{h}$ hash queries. Then, we have*

$$\begin{array}{c} Adv_{GAP,D}\left(A\right)\leq \frac{q_{h}^{2}}{\left|Hash\right|}+\frac{2q_{send}}{\left|D\right|}. \end{array}$$

**Proof of Theorem** **1.** To complete the proof, four experiments are constructed, where the first one simulates a real attack. For every experiment $Exp_{n}$, we use an event $Succ_{n}$ to denote the event in which the adversary successfully guesses the bit *b* from the Test query. ☐

**Experiment** $Exp_{0}.$ This experiment simulates an actual attack. According to definition, we have

(1) $${Adv}_{GAP,D}\left(A\right)=2Pr\left[Succ_{0}\right]-1.$$

**Experiment** $Exp_{1}.$ In this experiment, the oracles Execute, Send, Corrupt, Reveal, Test as in an actual attack are simulated. It can be seen that one cannot distinguish this experiment from the actual experiment. Thus,

(2) $$Pr\left[Succ_{1}\right]=Pr\left[Succ_{0}\right].$$

**Experiment** $Exp_{2}.$ All oracles considered in experiment ${\mathrm{Exp}}_{1}$ are also simulated in this experiment; however, all executions are halted where a collision occurs when simulating the Send and the *h* oracle. *A* issues Send to try to deceive the other participants into accepting a modified message. Simultaneously, it can query the *h* oracle to verify whether a hash collision exists. Since the messages transmitted in the network are all associated with a participant’s identity, a temporary secret random number and a long-lived key, and the authentication procedure only uses an XOR operation and a hash function, there is no other collision except hash collision. The probability of collision in the *h* oracle is at most $q_{h}^{2}/2|Hash|$ by the birthday paradox. Hence,

(3) $$|Pr\left[S{\mathrm{ucc}}_{1}\right]-Pr\left[S{\mathrm{ucc}}_{2}\right]|\leq \frac{q_{h}^{2}}{2|Hash|}.$$

**Experiment** $Exp_{3}.$ All oracles considered in experiment ${\mathrm{Exp}}_{2}$ are simulated in this experiment, in addition to stopping the stimulation of a Corrupt query to an OBU. Note that the information $B_{i}$, $C_{i}$,$D_{i}$, $y_{i}$, $Z_{i}$ and $P_{pub_{i}}$ stored in the OBU can be extracted by *A* when the $Corrupt(U_{i})$ query is issued. However, this information is useless to *A* for calculating the session key since it would also need the secret $A_{i}$, and it is difficult to derive $A_{i}$ from $B_{i}$ without also obtaining the user’s correct password $PW_{i}$ via the password attack. Hence, we obtain

(4) $$|Pr\left[S{\mathrm{ucc}}_{2}\right]-Pr\left[S{\mathrm{ucc}}_{3}\right]|\leq \frac{q_{send}}{\left|D\right|}.$$

In addition, we know that the adversary *A* can only win the game by guessing the bit *b* when querying the Test oracle because the adversary has no advantage. Therefore,

(5) $$Pr\left[S{\mathrm{ucc}}_{3}\right]=\frac{1}{2}.$$

From Equations (2) to (5), we have

$$\begin{array}{cc} |Pr\left[S{\mathrm{ucc}}_{0}\right]-\frac{1}{2}| & =|Pr\left[S{\mathrm{ucc}}_{0}\right]-Pr\left[S{\mathrm{ucc}}_{3}\right]|\\ & \leq |Pr\left[S{\mathrm{ucc}}_{0}\right]-Pr\left[S{\mathrm{ucc}}_{1}\right]|+|Pr\left[S{\mathrm{ucc}}_{1}\right]-Pr\left[S{\mathrm{ucc}}_{2}\right]|\\ & +|Pr\left[S{\mathrm{ucc}}_{2}\right]-Pr\left[S{\mathrm{ucc}}_{3}\right]|\\ & \leq \frac{q_{h}^{2}}{2|Hash|}+\frac{q_{send}}{\left|D\right|}. \end{array}$$

Therefore, from Equation (1), we get

$$\begin{array}{c} Adv_{GAP,D}\left(A\right)\leq \frac{q_{h}^{2}}{\left|Hash\right|}+\frac{2q_{send}}{\left|D\right|}. \end{array}$$

**Theorem** **2.** *Let G represent a group with a prime order p, and SCP denote the secure communication procedure presented in Figure 5. Let ℓ be the size of the identity space, |Hash| and |D| represent the range space of the hash function and the size of the password dictionary D. Finally, let A represent an adversary attacking the semantic security of the secure communication protocol with time-complexity at most t by issuing $q_{send}$ Send queries, $q_{exe}$ Execute queries and $q_{h}$ Hash queries. Then, we have:*

$$\begin{array}{cc} Adv_{SCP,D}\left(A\right) & \leq \frac{2(q_{send}+q_{exe})}{\ell }+\frac{q_{h}^{2}}{\left|Hash\right|}+\frac{{\left(q_{send}+q_{exe}\right)}^{2}}{p}+\frac{2q_{send}}{\left|D\right|}\\ & +2q_{h}Adv_{G}^{ECCDH}\left(t+\left(q_{send}+q_{exe}\right)t_{p}\right), \end{array}$$

*where $t_{p}$ denotes the time required to produce a point.*

**Proof of Theorem** **2.** To complete the proof, six experiments are constructed, where the first one simulates a real attack. For every experiment $Exp_{n}$, we use $Succ_{n}$ to denote the event in which the adversary successfully guesses the bit *b* from the Test query. ☐

**Experiment** $Exp_{0}.$ This experiment simulates an actual attack, which begins with the random selection of a secure key PSK. According to definition, we have

(6) $${Adv}_{SCP,D}\left(A\right)=2Pr\left[Succ_{0}\right]-1.$$

**Experiment** $Exp_{1}.$ In this experiment, the oracles Execute, Send, Corrupt, Reveal, and Test, as in the actual attack with a chosen random secure key PSK are simulated. It can be seen that one cannot distinguish this experiment from the actual experiment. Thus,

(7) $$Pr\left[Succ_{1}\right]=Pr\left[Succ_{0}\right].$$

**Experiment** $Exp_{2}.$ All oracles considered in experiment $Exp_{1}$ are also simulated in this experiment. In addition, we stop simulating the adversary to execute guessing attacks on the real identity of a participant. In this case, we have

(8) $$|Pr\left[Succ_{1}\right]-Pr\left[Succ_{2}\right]|⩽\frac{q_{send}+q_{exe}}{\ell }.$$

**Proof.**  Each participant’s real identity is always converted into an alias using a random number (i.e., $AID_{i}=ID_{i}⊕H\left(r_{i}P_{pub_{j}}\right)$). Therefore, the adversary cannot determine the participant’s real identity because every alias is different and there is nothing that can be used to verify the real identity. ☐

**Experiment** $Exp_{3}.$ All oracles considered in experiment ${\mathrm{Exp}}_{2}$ are also simulated in this experiment; however, all executions are halted where a collision occurs among $(AID_{i}$, $u_{i}$, $M_{1})$, $(AID_{j}$,$u_{j}$,$M_{2})$, and $\left(M_{3}\right)$. The probability of colliding in the *h* oracle is at most $q_{h}^{2}/2|Hash|$ by the birthday paradox. Similarly, the probability of colliding in the transcript is at most ${\left(q_{send}+q_{exe}\right)}^{2}/2p$, Consequently,

(9) $$|Pr\left[S\mathrm{uc}c_{2}\right]-Pr\left[S\mathrm{uc}c_{3}\right]|\leq \frac{q_{h}^{2}}{2|Hash|}+\frac{{\left(q_{send}+q_{exe}\right)}^{2}}{2p}.$$

**Experiment** $Exp_{4}.$ All oracles considered in as experiment ${\mathrm{Exp}}_{3}$ are simulated in this experiment, in addition to stopping the stimulation of a Corrupt query to an OBU. Note that the information $B_{i}$, $C_{i}$, $D_{i}$, $y_{i}$, $Z_{i}$, $P_{pub_{i}}$, and $E_{i}$ stored in the OBU can be extracted by *A* when the $Corrupt(U_{i})$ query is issued. However, this information is useless to *A* for calculating the session key since it would require the secure key PSK, a private key $x_{i}$ and a temporary secret random number, and it is difficult to derive PSK and $x_{i}$ from $E_{i}$ and $Z_{i}$ without obtaining the user’s correct password $PW_{i}$ via the password attack. Hence, we obtain

(10) $$|Pr\left[S\mathrm{uc}c_{3}\right]-Pr\left[S\mathrm{uc}c_{4}\right]|\leq \frac{q_{send}}{\left|D\right|}.$$

**Experiment** $Exp_{5}.$ In this experiment, we use the private oracle $h^{\prime }$ in place of the oracle *h* for computing *k* as shown in Table 3, such that the session key is totally independent of *h*. More precisely, one obtains $k=h^{\prime }(T∥R∥P_{pub_{i}}∥P_{pub_{j}})$ in Execute queries. Therefore, the experiments $Exp_{4}$ and $Exp_{5}$ are indistinguishable except for the occurrence of the following event $AskH_{6}$: *A* issues queries to *h* on $T∥R∥P_{pub_{i}}∥P_{pub_{j}}∥s$, i.e., the value $T∥R∥P_{pub_{i}}∥P_{pub_{j}}∥ECCDH\left(T,P_{pub_{j}}\right)+ECCDH\left(R,P_{pub_{i}}\right)$. In addition, regardless of the *b* value that is chosen to be used in a Test query, the response is independent for all sessions since it is a random number. Therefore,

(11) $$Pr\left[S\mathrm{uc}c_{5}\right]=\frac{1}{2}.$$

**Experiment** $Exp_{6}.$ The execution of the random self-reducibility of the elliptic curve computational Diffie–Hellman assumption given an ECCDH instance (A,B) is simulated in this experiment. We randomly select $\alpha ,\beta ,\gamma ,\varphi \in Z_{p}^{*}$, and let T=αA−PSK·P, R=βA−PSK·P, $P_{pub_{i}}=\gamma B$, and $P_{pub_{j}}=\varphi B$ . Note that $AskH_{6}$ means that a query *h* on T||R||Y has been issued by *A*, where $Y=ECCDH\left(T,P_{pub_{j}}\right)+ECCDH\left(R,P_{pub_{i}}\right)$. Indeed, $\mathrm{Pr}\left[AskH_{6}\right]=\mathrm{Pr}\left[S\mathrm{uc}c_{6}\right]$ . We have:

$$ECCDH\left(T,P_{pub_{j}}\right)=\alpha \varphi \cdot ECCDH\left(A,B\right)-\varphi PSK\cdot B,$$

$$ECCDH\left(R,P_{pub_{i}}\right)=\beta \gamma \cdot ECCDH\left(A,B\right)-\gamma PSK\cdot B.$$

Therefore,

$$ECCDH\left(T,P_{pub_{j}}\right)+ECCDH\left(R,P_{pub_{i}}\right)=\left(\alpha \varphi +\beta \gamma \right)\cdot ECCDH\left(A,B\right)-\left(\varphi +\gamma \right)PSK\cdot B.$$

If *A* knows the session key *k* constructed by (αA,βA,PSK·P,γB,φB), it must have issued queries to *h* on $T∥R∥P_{pub_{i}}∥P_{pub_{j}}∥s$ that was recorded in the list $\Lambda_{h}$. Therefore, we can conclude that

(12) $$\mathrm{Pr}\left[Succ_{6}\right]\leq q_{h}Adv_{G}^{ECCDH}\left(t+\left(q_{send}+q_{exe}\right)t_{p}\right).$$

From Equations (7) to (12), we have

$$\begin{array}{cc} |Pr\left[S{\mathrm{ucc}}_{0}\right]-\frac{1}{2}| & =|Pr\left[S{\mathrm{ucc}}_{0}\right]-Pr\left[S{\mathrm{ucc}}_{5}\right]|\\ & \leq |Pr\left[S{\mathrm{ucc}}_{0}\right]-Pr\left[S{\mathrm{ucc}}_{1}\right]|+|Pr\left[S{\mathrm{ucc}}_{1}\right]-Pr\left[S{\mathrm{ucc}}_{2}\right]|+|Pr\left[S{\mathrm{ucc}}_{2}\right]-Pr\left[S{\mathrm{ucc}}_{3}\right]|\\ & +|Pr\left[S{\mathrm{ucc}}_{3}\right]-Pr\left[S{\mathrm{ucc}}_{4}\right]|+|Pr\left[S{\mathrm{ucc}}_{4}\right]-Pr\left[S{\mathrm{ucc}}_{5}\right]|\\ & \leq \frac{q_{send}+q_{exe}}{\ell }+\frac{q_{h}^{2}}{2|Hash|}+\frac{{\left(q_{send}+q_{exe}\right)}^{2}}{2p}+\frac{q_{send}}{\left|D\right|}\\ & +q_{h}Adv_{G}^{ECCDH}\left(t+\left(q_{send}+q_{exe}\right)t_{p}\right). \end{array}$$

Therefore, from Equation (6), we get

$$\begin{array}{cc} Adv_{SCP,D}\left(A\right) & \leq \frac{2(q_{send}+q_{exe})}{\ell }+\frac{q_{h}^{2}}{\left|Hash\right|}+\frac{{\left(q_{send}+q_{exe}\right)}^{2}}{p}+\frac{2q_{send}}{\left|D\right|}\\ & +2q_{h}Adv_{G}^{ECCDH}\left(t+\left(q_{send}+q_{exe}\right)t_{p}\right). \end{array}$$

#### 6.2.2. Informal Security Analysis

##### Confidentiality of Session Key

In our proposed scheme, when an authentication, secure communication or key update procedure is performed, a session key is generated using two random numbers chosen by the participants. Then, the generated key is used to ensure a secure communication. Moreover, the random numbers used to generate each session key are different. Therefore, it is difficult for an adversary *A* to successfully guess the session key or derived it from the communicated messages.

##### Anonymity

In our proposed scheme, to preserve users’ privacy, the original identity of every participant is converted into an alias via an XOR operation with a hash that takes a random number $r_{i}$ as an input (i.e., $AID_{i}=ID_{i}⨁h\left(r_{i}\right)$, $AID_{i}=ID_{i}⨁h\left(r_{i}P_{pub_{j}}\right)$). Therefore, an adversary *A* cannot determine a user’s original identity without the random number $r_{i}$ or the private key $x_{j}$ even if *T* has been obtained because of the hardness of the ECCDH problem in *G*.

##### Unlinkability

In our proposed scheme, the original identities of the participants are not transmitted over the unsecure network; instead, every participant’s identity is converted into an alias. Moreover, the authentication, secure communication and key update phases are independent of each other. In addition, after every authentication procedure performed by $OBU_{i}$, the value $C_{i}$ updates itself. Therefore, for two or more authentication messages that are sent by the same user, the adversary *A* cannot determine whether they have the same origin. Thus, *A* cannot trace the location of a user by intercepting messages.

##### Resistance to Impersonation Attack

In the authentication procedure of our improved scheme, if an adversary wishes to impersonate $OBU_{i}$, it must obtain both the $A_{i}$ and $ID_{i}$ of $OBU_{i}$. Otherwise, it cannot compute a valid authentication request, since the original identity of $OBU_{i}$ is converted into an alias via an XOR operation with a random number $r_{i}$ chosen by itself and this random number $r_{i}$ is hidden by its $A_{i}$. Moreover, the adversary can successfully impersonates $OBU_{i}$ only by correctly guessing the random number, which is difficult because the random number is reselected with each authentication. Furthermore, in the secure communication procedure, the original identity of $OBU_{i}$ is also converted into an alias with a random number $r_{i}$($AID_{i}=ID_{i}⨁h\left(r_{i}P_{pub_{j}}\right)$). The adversary cannot successfully impersonate the OBU since the random number cannot be guessed.

##### Resistance to Internal Attack

In our proposed scheme, an internal attack refers to the case in which the owner of a vehicle, who possesses the common secure key PSK, attempts to reveal the session key for a communication channel. Under our improved scheme, in the secure communication procedure, even if the adversary can intercept all exchanged messages, $(AID_{i}$, $u_{i})$ of $OBU_{i}$ and $(AiD_{j}$, $u_{j})$ of $OBU_{j}$ and compute *T* and *R* using the secure key PSK, it cannot determine the user’s original identity or compute the session key *k* under the assumption of the hardness of ECCDH problem in *G*.

### 6.3. Performance Analysis

In our proposed scheme, the general authentication procedure is based only on an XOR operation and a hash function; thus, the computation cost is low. To demonstrate the performance of the proposed scheme, we compare the the critical secure communication procedure with the existing two-party secure communication schemes with session key agreement [6,11,12,15,16]. Next, we implement our scheme based on cryptographic libraries and present a concrete comparison of execution times. Then, we compare the security features of these schemes. Some notations are defined as follows for convenience:

*T_h_* : The execution time of a hash function operation.
*T_bp_* : The execution time of a bilinear pairing operation.
*T_mul_* : The execution time of an ECC-based scalar point multiplication operation.
*T_add_* : The execution time of an ECC-based scalar point addition operation.

The detailed comparison is presented in Table 4, where the middle and right columns list the complexity and total execution time, respectively, of each scheme. The transmission time is not considered in the comparison since it depends on the actual characteristics of the network, not the scheme. All operations listed in Table 4 were implemented using the OpenSSL library and the JPBC library, and the experiments were conducted on a Windows 7 PC (Samsung Electronics, Hwaseong, Korea) equipped with an Intel(R) Core(TM) i7-6500U CPU (Santa Clara, CA, USA).

**Figure 6:**
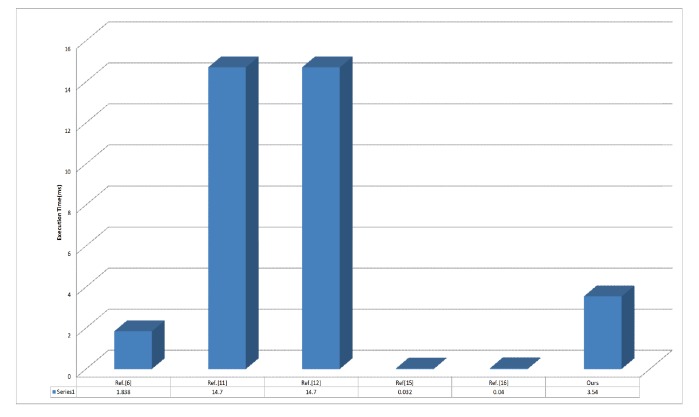
Execution time(ms) of different authentication protocols.

As seen in Table 5 and Figure 6, the execution time of our scheme is less than those of some other schemes [11,12]. Although the execution times of Chuang–Lee’s scheme and Kumari’s scheme are less than that of our scheme, their schemes fail to resist internal attack because the participants’aliases depend only on a random number that is hidden by PSK as shown in Table 6. Therefore, a trusted vehicle can reveal a participant’s real identity because it holds PSK. Meanwhile, because Porambage’s scheme uses certificates for authentication, the unlinkability of messages cannot be preserved, and a user’s anonymity can be violated. Therefore, our proposed scheme is a preferable solution for secure communication in vehicle sensor networks compared with the existing similar schemes presented in [6,11,12,15,16].

## 7. Conclusions

With the emergence of intelligent transportation, the security of vehicle sensor networks is attracting attention from individuals and vehicle manufacturers, and privacy preservation in communication over vehicle sensor networks has become a critical issue. In this paper, we have demonstrated that Chuang and Lee’s TEAM scheme exists the linkability of messages in the authentication protocol; thus, a malicious vehicle can track a driver by intercepting transmitted message. Simultaneously, TEAM scheme can suffer the internal attack in the secure communication protocol; thus, a malicious trusted vehicle can compute the real identity of a user and the session key. To address this shortcoming, an improved authentication scheme based on elliptic curves for better performance and security has been constructed, in which the difficulty of deriving real identities arises from the need to solve an elliptic curve discrete logarithm problem. In this way, privacy preservation is achieved since the real identities of users are protected. The correctness of our proposed scheme has been proven using BAN logic, and a rigorous security proof has been provided based on the random oracle model. In future work, elliptic curves based authentication schemes involving three parities will be investigated.

## Figures and Tables

**Table 1:** The notations.

Notation	Definitions
*x*	A private key for the AS
$x_{i}$	A private key for user *i*
PSK	A pre-shared secure key set among the LEs and the AS
$ID_{i}$	The identification code for entity *i*
$PW_{i}$	The password for user *i*
$AID_{i}$	The alias for entity *i*
*h*	A secure hash function
⊕	The XOR operator
∥	The combination of strings
$P_{pub_{i}}$	A public key for user *i* and $P_{pub_{i}}=x_{i}P$
*p*	A secure large prime
$E(F_{p})$	A secure elliptic curve
*P*	The primitive generator for *G*
*G*	The subgroup of $E(F_{p})$ with order *p*
$Z_{p}^{*}$	The set consisting of all primes in {0,1,…,p−1}
$r,y\in_{R}Z_{p}^{*}$	An element selected randomly from $Z_{p}^{*}$
$SK_{ij}$	A session key between entity *i* and entity *j*
$MSG_{KU}$	A key update message

**Table 2:** Symbol and description of BAN logic.

Symbol	Description
P∣≡X	Entity *P* trusts opinion *X*
P◃X	Entity *P* sees opinion *X*, or *P* holds *X*
P∣∼X	Entity *P* has said opinion *X*
P∣⇒X	Entity *P* completely control over *X*
♯(X)	*X* is fresh
$\frac{Rule1}{Rule2}$	Rule2 comes from Rule1
$\overset{k}{\mapsto }P$	*k* is the public key of entity *P*
$P\overset{k}{⟷}Q$	*k* is a secret key or information between *P* and *Q*
${\left\{X\right\}}_{PSK}$	*X* is encrypted by key *K*

**Table 3:** Simulation of random oracles *h* and $h^{\prime }$.

A hash query h(m) (resp.$h^{\prime }$) that matches a record $\left(m,r^{\prime }\right)$ in the list $\Lambda_{h}$ (resp.$\Lambda_{h^{\prime }}$), returns $r^{\prime }$.
Otherwise, it chooses a random number *r*, adds the record (m,r) to the list $\Lambda_{h}$ (resp.$\Lambda_{h^{\prime }}$), and returns *r*.

**Table 4:** The execution time of basic operation.

Operation	$T_{h}$	$T_{\mathrm{mul}}$	$T_{\mathrm{bp}}$	$T_{\mathrm{add}}$
Execution time (ms)	0.004	0.326	6.28	0.038

**Table 5:** Comparison of efficiency.

Scheme	Computation Cost	Computation Time (ms)
Reference [6]	$4T_{h}+4T_{mul}+2T_{add}$	≈1.838
Reference [11]	$10T_{h}+6T_{mul}+2T_{bp}+4T_{add}$	≈14.7
Reference [12]	$10T_{h}+6T_{mul}+2T_{bp}+4T_{add}$	≈14.7
Reference [15]	$8T_{h}$	≈0.032
Reference [16]	$10T_{h}$	≈0.04
Proposed	$12T_{h}+10T_{mul}+6T_{add}$	≈3.54

**Table 6:** Comparison of security features.

Security Threats and Scheme	Ref. [6]	Ref. [11]	Ref. [12]	Ref. [15]	Ref. [16]	Proposed
Provides user anonymity	×	×	×	×	×	√
Resistance to user traceability attack	×	×	×	×	×	√
Resistance to impersonation attack	√	×	√	√	√	√
Resist inside attack	√	√	√	×	×	√
Unlinkability of message	×	√	√	×	√	√
